# Spondyloarthropathy: diagnostic imaging criteria for the detection of sacroiliitis

**DOI:** 10.1590/0100-3984.2015-0211

**Published:** 2017

**Authors:** Moacir Ribeiro de Castro Jr., Sonia de Aguiar Vilela Mitraud, Marina Celli Francisco, Artur da Rocha Corrêa Fernandes, Eloy de Ávila Fernandes

**Affiliations:** 1 Doctoral Student in the Graduate Program in Clinical Radiology, Collaborating Physician in the Department of Diagnostic Imaging of the Escola Paulista de Medicina da Universidade Federal de São Paulo (EPM-Unifesp), São Paulo, SP, Brazil.; 2 PhD, Coordinator of the Computed Tomography Sector at the Hospital São Paulo - Escola Paulista de Medicina da Universidade Federal de São Paulo (EPM-Unifesp), São Paulo, SP, Brazil.; 3 MD, Radiologist at Clínica IMED - Clínica Médica e Diagnóstico por Imagem, Curitibanos, SC, Brazil.; 4 PhD, Associate Professor in the Department of Diagnostic Imaging of the Escola Paulista de Medicina da Universidade Federal de São Paulo (EPM-Unifesp), São Paulo, SP, Brazil.; 5 PhD, Advising Professor for the Graduate Program in Clinical Radiology of the Escola Paulista de Medicina da Universidade Federal de São Paulo (EPM-Unifesp), São Paulo, SP, Brazil.

**Keywords:** Sacroiliitis, Magnetic resonance imaging, Spondyloarthropathies, Ankylosing spondylitis, Computed tomography, Radiography

## Abstract

Diagnostic imaging is crucial to the diagnosis and monitoring of spondyloarthropathies. Magnetic resonance imaging is the most relevant tool for the early detection of sacroiliitis, allowing the institution of therapeutic strategies to impede the progression of the disease. This study illustrates the major criteria for a magnetic resonance imaging-based diagnosis of spondyloarthropathy. The cases selected here present images obtained from the medical records of patients diagnosed with sacroiliitis over a two-year period at our facility, depicting the active and chronic, irreversible forms of the disease. Although computed tomography and conventional radiography can also identify structural changes, such as subchondral sclerosis, erosions, fat deposits, and ankylosis, only magnetic resonance imaging can reveal active inflammatory lesions, such as bone edema, osteitis, synovitis, enthesitis, and capsulitis.

## Abstract (pt)

A avaliação por imagem é fundamental para o diagnóstico e acompanhamento clínico dos pacientes com espondiloartropatias. A ressonância magnética é o método de imagem mais importante para a detecção precoce de sacroileíte, permitindo a instituição de terapias que podem impedir a progressão da doença. Este estudo ilustra os principais critérios de ressonância magnética na definição de sacroileíte nas espondiloartropatias, com imagens selecionadas dos prontuários dos pacientes diagnosticados no nosso serviço, demonstrando tanto os achados da doença em atividade como as alterações crônicas de caráter irreversível. Embora a tomografia computadorizada e a radiografia convencional possam identificar lesões estruturais crônicas, tais como esclerose subcondral, erosões, depósitos de gordura e anquilose, apenas a ressonância magnética é capaz de demonstrar lesões inflamatórias ativas, tais como edema ósseo, osteíte, sinovite, entesite e capsulite.

## INTRODUCTION

Seronegative spondyloarthropathies include a group of chronic systemic inflammatory diseases, characterized by the absence of rheumatoid factor in serum, common clinical findings (such as inflammatory arthritis and enthesitis), inflammatory low back pain, and the presence of human leukocyte antigen^(1,2)^. This group of diseases includes ankylosing spondylitis, psoriatic arthritis, reactive arthritis, inflammatory enteropathic arthritis, and undifferentiated spondyloarthropathy.

Patients with seronegative spondyloarthropathies typically present early clinical manifestations in the sacroiliac joints. These diseases evolve slowly, and there are no specific biochemical markers that demonstrate their activity. Therefore, in clinical practice, imaging, more precisely magnetic resonance imaging (MRI), typically forms the basis for the diagnosis and evaluation of sacroiliitis^(3-5)^.

MRI has become an integral part of the diagnostic process because it is the most relevant imaging method for the classification and monitoring of spondyloarthropathies. Radiography also plays an important role in the diagnosis of sacroiliitis. However, these diseases are typically not detected until three to seven years after their onset. In addition, X-ray and computed tomography allow structural changes to be identified only when the damage has already become irreversible^(5,6)^.

## ACTIVE INFLAMMATORY LESIONS

### Bone marrow edema/osteitis

**Figure 1:**
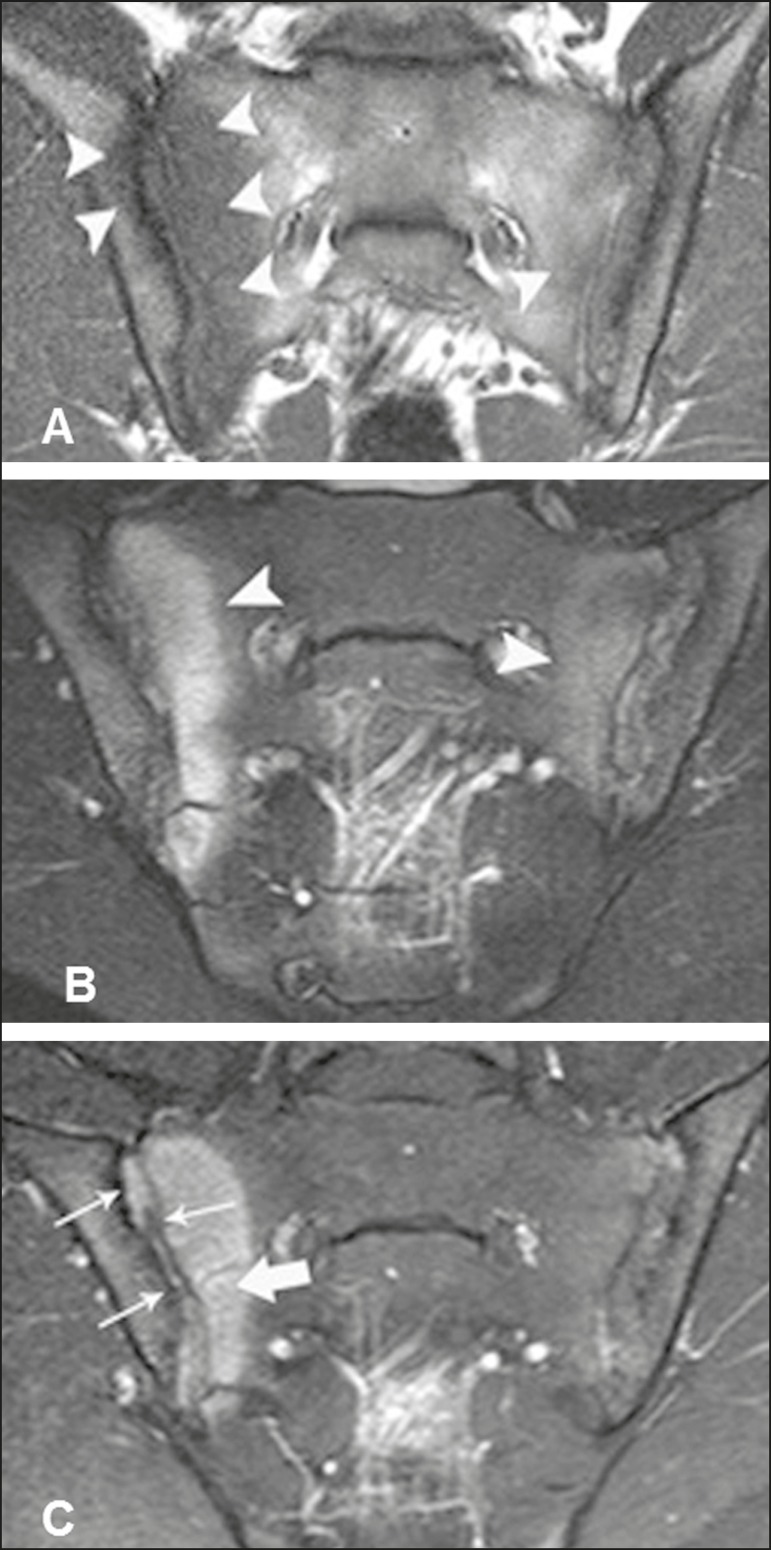
22-year-old male patient diagnosed with seronegative spondyloarthropathy 6 years prior. **A,B:** Fast spin-echo T1-weighted and STIR sequences showing subchondral bone edema (arrowheads). **C:** Intravenous contrast-enhanced T1-weighted sequences with fat saturation showing periarticular and subchondral osteitis with bone marrow enhancement (thick arrow). Synovitis with right sacroiliac intra-articular enhancement (thin arrows).

Bone marrow edema/osteitis appears as an area of low signal intensity on T1-weighted images and high signal intensity in short-tau inversion-recovery (STIR) sequences or equivalent liquid-sensitive sequences, located in the subchondral bone marrow (Figure 1). The edema should be easily characterized, with a signal similar to that of the cerebrospinal fluid. When present, bone marrow edema is indicative of active sacroiliitis, not being pathognomonic of spondyloarthropathies, and can be related to other conditions, such as alterations caused by mechanical overload. It should be noted that, in general, those alterations are not restricted to a single image and when accompanied by structural findings, such as subchondral sclerosis and bone erosion, allow a better diagnostic determination if taken together with the clinical and laboratory data. A finding of bone marrow located among the sacral foramina can be used as a reference for normality.

### Synovitis, enthesitis, and capsulitis

On intravenous paramagnetic contrast-enhanced T1-weighted images with fat suppression, synovitis is characterized by enhancement in the synovial part of the sacroiliac joint. In STIR sequences, the hyperintense signal within the synovial portion of the sacroiliac joint precludes good differentiation between synovitis and physiologic fluid on the joint^(6)^.

**Figure 2:**
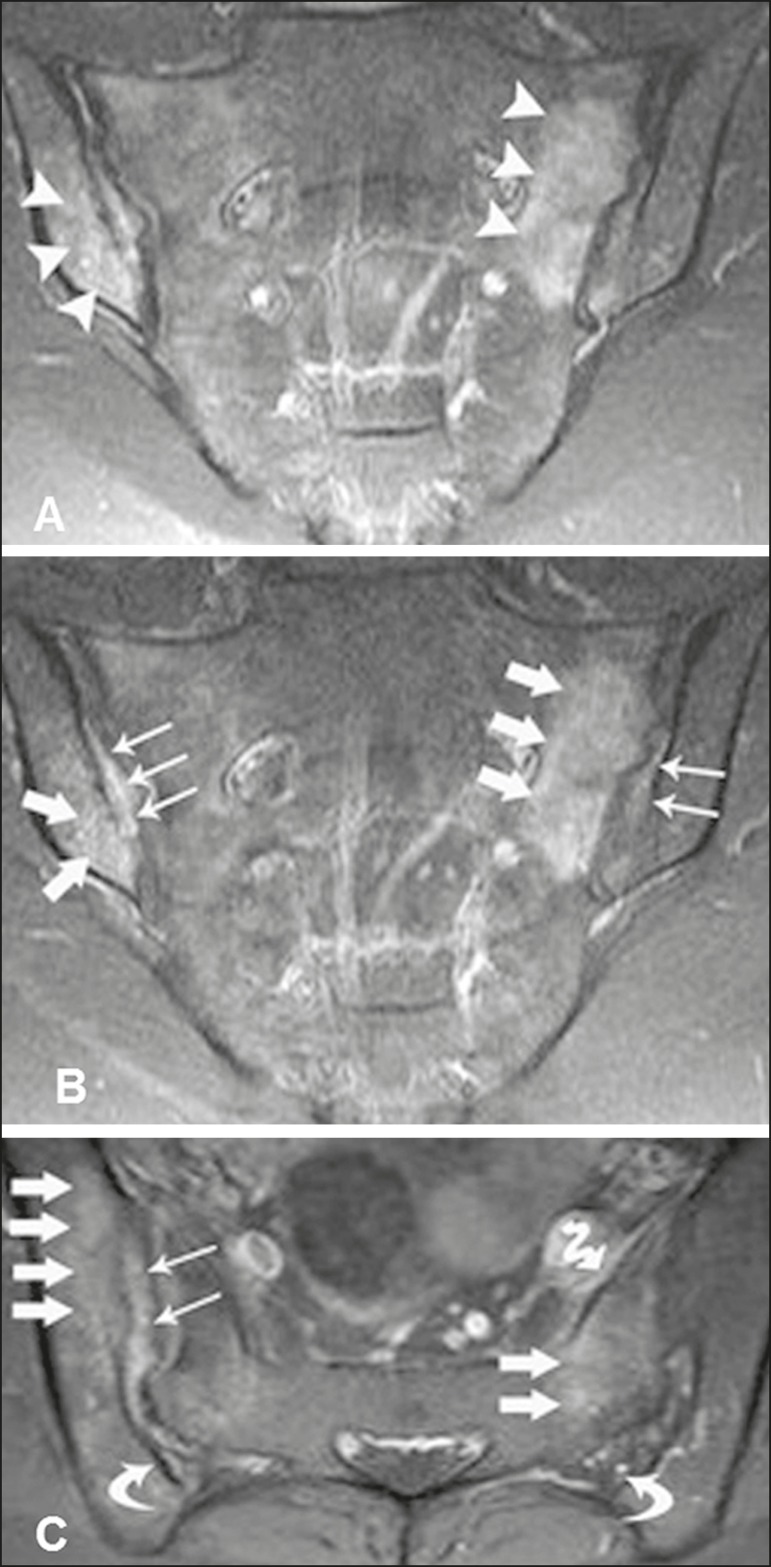
28-year-old female patient diagnosed with seronegative spondyloarthropathy 8 years prior. **A:** Coronal STIR sequence showing subchondral edema (arrowheads). **B,C:** Intravenous contrast-enhanced coronal and axial T1-weighted sequences with fat saturation, showing osteitis (thick arrows), synovitis characterized by intra-articular enhancement (thin arrows), enthesitis with enhancement in the ligamentous compartment of the joint (curved arrows) and capsulitis with pericapsular enhancement on the left (serpentine arrow).

Enthesitis is defined as an area of high signal intensity in STIR sequences, or as an area of enhancement in contrast-enhanced sequences, at the ligament insertion sites within the retroarticular space, potentially extending to the bones and soft tissues^(6)^ (Figure 2).

**Figure 3:**
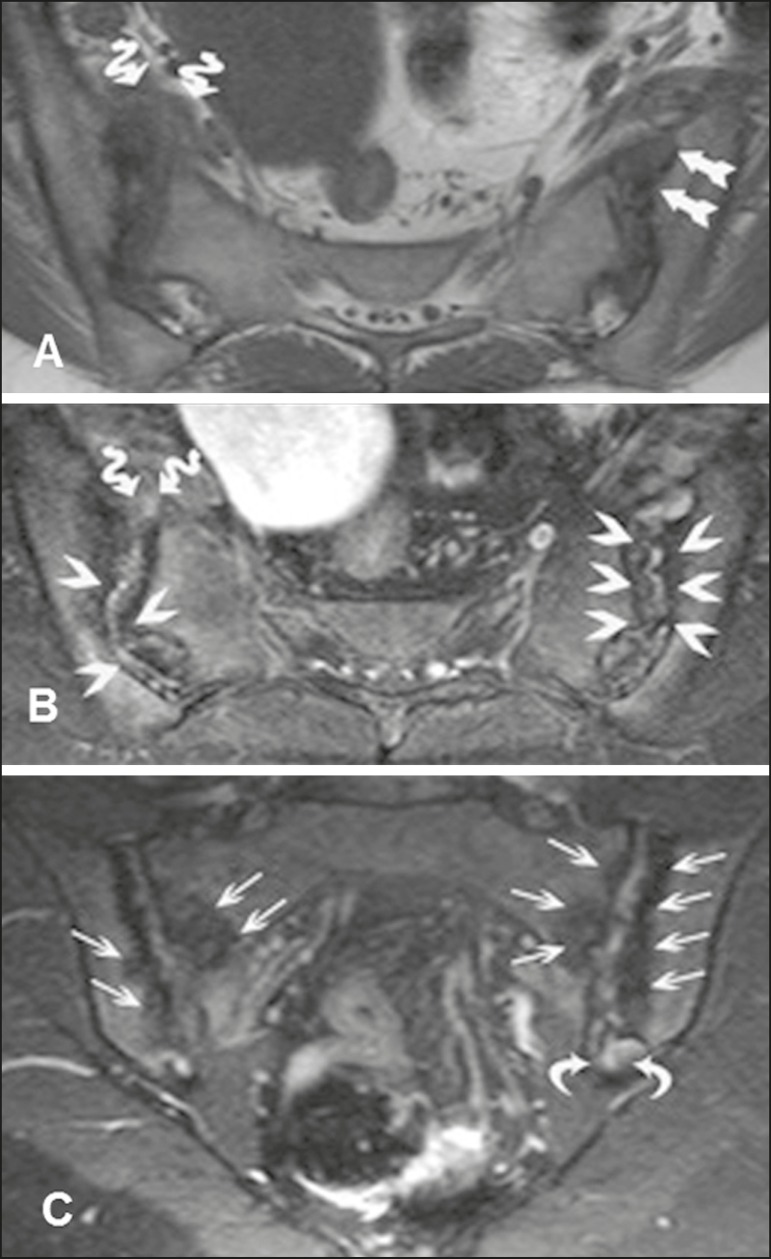
41-year-old female patient diagnosed with seronegative spondyloarthropathy 3 years prior. **A:** Axial fast spin-echo T1-weighted sequence showing periarticular bone erosion (straight arrows) and thickening of the right joint capsule (serpentine arrows). B: Axial STIR sequence showing pseudo-widening of the joint spaces (arrowheads). Thickening and edema of the right joint capsule (serpentine arrows), characteristic of capsulitis. **C:** Intravenous contrast-enhanced coronal T1-weighted sequence with fat suppression, showing areas of subchondral bone sclerosis (thin arrows); intra-articular enhancement in the left posterior fibrous region, indicative of enthesitis (curved arrows).

Capsulitis presents signaling characteristics similar to those of enthesitis, although the former affects the anterior and posterior portions of the joint capsule^(6)^ (Figure 3).

## STRUCTURAL CHANGES

Structural changes, which indicate previous inflammatory events, can be identified by MRI, X-ray, or computed tomography. Structural changes in sacroiliitis include subchondral sclerosis, bone erosion, fatty deposits, and bone bridges/ankylosis^(7)^.

### Subchondral sclerosis

In T1-weighted sequences, subchondral sclerosis is characterized by a hypointense signal that extends for at least 5 mm into the sacroiliac joint space. Mild forms of subchondral sclerosis can be seen in healthy individuals^(6)^.

### Bone erosion

**Figure 4:**
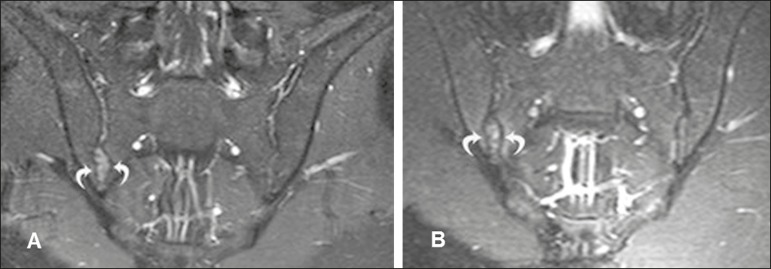
17-year-old male patient recently diagnosed with seronegative spondyloarthropathy. **A,B:** Intravenous contrast-enhanced coronal STIR and T1-weighted sequences with fat saturation, showing edema in the fibrous region of the right sacroiliac joint, characteristic of enthesitis.

Bone erosion is defined as focal lesions at the margin of the articular cartilage. The confluence of erosion sites is visualized as pseudo-widening of the sacroiliac joints^(6)^ (Figure 4).

### Periarticular fat deposits

**Figure 5:**
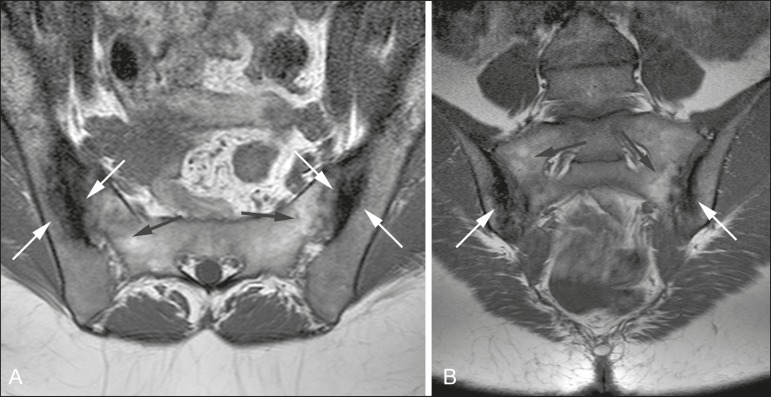
49-year-old female patient diagnosed with seronegative spondyloarthropathy 7 years prior. **A,B:** Axial fast spin-echo T1-weighted sequence and coronal T1-weighted sequence showing subchondral sclerosis (white arrows) and fat deposits (black arrows).

Fat deposits result from the esterification of fatty acids, which leads to inflammation, usually in the periarticular bone marrow. This is a nonspecific finding and is characterized by high signal intensity on T1-weighted images, indicating an area of previous inflammation^(5)^ (Figure 5).

### Bone bridges/ankylosis

**Figure 6:**
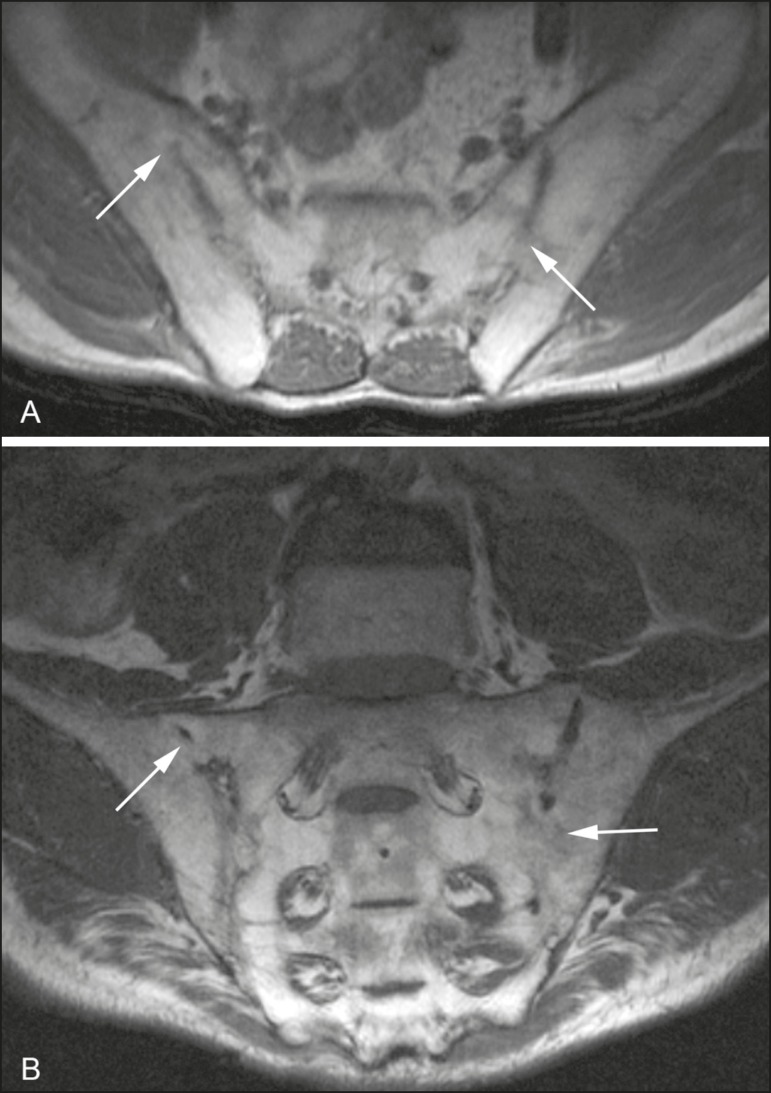
61-year-old male patient diagnosed with seronegative spondyloarthropathy 11 years prior. **A,B:** Axial fast spin-echo T1-weighted sequence and coronal fast spin-echo T1-weighted sequence showing bone bridges/ankylosis (arrows).

Bone bridges/ankylosis appear as areas of low signal intensity in all sequences and can show a bone marrow-like signal when fusion is complete. In addition, the joint space can become undefined^(6)^ (Figure 6).

## CRITERIA FOR DEFINING SACROILIITIS ACTIVITY

Bone edema and osteitis may indicate active sacroiliitis if seen at a typical site (periarticular or subchondral) on two consecutive MR scans. In cases in which bone edema is found under the conditions described above, contrast injection can be dispensed with. In isolation, synovitis, enthesitis, and capsulitis do not confirm active sacroiliitis, although they can facilitate the making of the correct diagnosis when considered together with bone edema or osteitis, even if they are present in the same image. The examination should be considered negative if the bone edema is not obvious^(7)^.

Althoff et al. reported that contrast-enhanced MRI examinations are useful in the early diagnosis of inflammatory sacroiliitis and could help boost the confidence of inexperienced radiologists. However, in patients with pre-existing disease or in patients undergoing follow-up examinations, the use of contrast agents can be dispensed with^(7)^.

Diffusion-weighted MRI seems to be an alternative means of using contrast agents in seronegative spondyloarthropathies. Diffusion-weighted imaging shows higher apparent diffusion coefficient values in patients with areas of bone marrow edema than in those with mechanical low back pain, although further studies are needed in order to determine the relevance of this technique in imaging studies of seronegative spondyloarthropathies^(8)^.

Knowledge of the diagnostic criteria for disease activity is essential for the radiologist, in order to facilitate the early diagnosis and treatment, as well as the follow-up, of spondyloarthropathies, thus reducing the associated morbidity and improving the quality of life of the affected patients.

The objective of the present study was to disseminate the MRI diagnostic criteria for sacroiliitis to radiologists, orthopedists, and rheumatologists. Knowledge of those criteria is fundamental to making the diagnosis in the acute, chronic inactive, and chronic active phases of inflammatory activity.
